# Were Frailty Identification Criteria Created Equal? A Comparative Case Study on Continuous Non-Invasively Collected Neurocardiovascular Signals during an Active Standing Test in the Irish Longitudinal Study on Ageing (TILDA)

**DOI:** 10.3390/s24020442

**Published:** 2024-01-11

**Authors:** Feng Xue, Silvin Knight, Emma Connolly, Aisling O’Halloran, Morgana Afonso Shirsath, Louise Newman, Eoin Duggan, Rose Anne Kenny, Roman Romero-Ortuno

**Affiliations:** 1 Discipline of Medical Gerontology, School of Medicine, Trinity College Dublin, D02 PN40 Dublin, Ireland; 2 The Irish Longitudinal Study on Ageing (TILDA), Trinity College Dublin, D02 PN40 Dublin, Ireland

**Keywords:** frailty index, frailty phenotype, clinical frailty scale, active stand, continuous physiological monitoring, neurovascular, cardiovascular, statistical parametric mapping, frailty comparison

## Abstract

Background: In this observational study, we compared continuous physiological signals during an active standing test in adults aged 50 years and over, characterised as frail by three different criteria, using data from The Irish Longitudinal Study on Ageing (TILDA). Methods: This study utilised data from TILDA, an ongoing landmark prospective cohort study of community-dwelling adults aged 50 years or older in Ireland. The initial sampling strategy in TILDA was based on random geodirectory sampling. Four independent groups were identified: those characterised as frail only by one of the frailty tools used (the physical Frailty Phenotype (FP), the 32-item Frailty Index (FI), or the Clinical Frailty Scale (CFS) classification tree), and a fourth group where participants were not characterised as frail by any of these tools. Continuous non-invasive physiological signals were collected during an active standing test, including systolic (sBP) and diastolic (dBP) blood pressure, as well as heart rate (HR), using digital artery photoplethysmography. Additionally, the frontal lobe cerebral oxygenation (Oxy), deoxygenation (Deoxy), and tissue saturation index (TSI) were also non-invasively measured using near-infrared spectroscopy (NIRS). The signals were visualised across frailty groups and statistically compared using one-dimensional statistical parametric mapping (SPM). Results: A total of 1124 participants (mean age of 63.5 years; 50.2% women) were included: 23 were characterised as frail only by the FP, 97 by the FI, 38 by the CFS, and 966 by none of these criteria. The SPM analyses revealed that only the group characterised as frail by the FI had significantly different signals (*p* < 0.001) compared to the non-frail group. Specifically, they exhibited an attenuated gain in HR between 10 and 15 s post-stand and larger deficits in sBP and dBP between 15 and 20 s post-stand. Conclusions: The FI proved to be more adept at capturing distinct physiological responses to standing, likely due to its direct inclusion of cardiovascular morbidities in its definition. Significant differences were observed in the dynamics of cardiovascular signals among the frail populations identified by different frailty criteria, suggesting that caution should be taken when employing frailty identification tools on physiological signals, particularly the neurocardiovascular signals in an active standing test.

## 1. Introduction

Frailty is a distinctive health state related to the acceleration of the biological ageing process in which multiple body systems disproportionately lose their in-built reserves; as a result, older people living with frailty are at higher risk of adverse health outcomes when their physiology is suddenly challenged [1,2]. Frailty is increasingly recognised as an important concept in medicine because it has significant health implications for older adults’ wellbeing at multiple levels, including the physical, cognitive, social, and emotional levels [3,4]. Many studies have shown that older people living with frailty are at higher risk of falls, hospitalisation, worsening disability, and premature mortality [5,6].

The central tenet of frailty is the potential for serious adverse outcomes after a seemingly minor stressor event or challenge [7]. The early detection of frailty is therefore of paramount importance because evidence has shown that targeted interventions may be able to increase physiological resilience in older adults [8]. While the full assessment and individualised management of frailty typically encompass a comprehensive multidisciplinary evaluation of an individual’s physical, cognitive, and social function [9,10], many tools have been developed to rapidly identify frailty and hence help healthcare workers prioritise those who need comprehensive geriatric assessment [11]. Since different frailty identification tools incorporate different elements of the geriatric assessment, they have been shown to capture different morbidity and functional profiles (e.g., degree of disability) and long-term risks (e.g., mortality) [12,13], but there is a paucity of data as to how different frailty tools capture differences in continuous physiological signals during stressor challenges [14].

A physiological stressor that people experience multiple times on a daily basis is the orthostatic challenge, which requires the individual to counteract the physiological demands imposed by the act of standing up quickly from a lying or sitting position [15]. Upon shifting to an upright posture, the gravity-induced accumulation of blood (i.e., blood pooling) in the compliant distensible veins of the abdomen and lower extremities [16] causes a reduction in the central venous pressure, leading to declines in the venous return, stroke volume, and arterial pressure [17]. The primary response mechanism to orthostatic stress involves the withdrawal of vagal activity and the activation of the sympathetic nervous system through the baroreflex, resulting in elevated heart rate and blood pressure [18]. In addition, there are dynamic cerebrovascular changes to ensure that the oxygen supply to the brain remains as constant as possible [19]. Some of these complex real-time cardiovascular and neurovascular adaptations can be continuously measured using non-invasive biosensors, e.g., as regards fluctuations in the blood pressure and heart rate and the degree of brain tissue oxygenation.

Abnormal physiological adaptations to standing, often referred to as neurocardiovascular instability (NCVI) [20], have been described in older people using a single frailty identification tool [21]. However, it remains unknown to date whether different frailty criteria render differently in neurocardiovascular signals during an orthostatic stress test. Consequently, a knowledge gap remains in the applicability of frailty identification criteria to physiological signals. In this study, we compared three well-established frailty identification criteria by examining the dynamics of the cardiovascular and neurovascular responses during an active standing test, using the non-invasively collected neurocardiovascular data from The Irish Longitudinal Study on Ageing (TILDA), with the aim of raising the awareness that different frailty classification criteria can manifest differently in physiological signals.

## 2. Materials and Methods

### 2.1. Study Population

TILDA is a landmark prospective cohort study of community-dwelling adults aged 50 years or older in Ireland. Data were collected at each wave via computer-aided personal interviewing and self-completed questionnaire, and at waves 1, 3, and 6 by a comprehensive centre- or home-based health assessment. In this study, we used the data of TILDA participants who completed the health centre assessment at wave 3 (2014–2015). Briefly, the sampling methods of TILDA were based at wave 1 on random geodirectory sampling and are detailed elsewhere [22,23,24]. Of note, the active standing test was not available in the home health assessment [24]. Approval for ethical considerations was obtained from the Faculty of Health Sciences Research Ethics Committee at Trinity College Dublin, Ireland. Written informed consent was provided from all participants, and the research was conducted in adherence to the principles outlined in the Declaration of Helsinki.

### 2.2. Frailty Identification Tools

For comparison of continuous orthostatic physiologies, we identified four mutually exclusive groups: groups characterised as frail by only one tool (but not the others), including the physical Frailty Phenotype (FP) [25], Frailty Index (FI) [26], and Clinical Frailty Scale (CFS) classification tree [27], and a fourth group, where participants were not considered frail according to any of these tools. The FP, CFS [28], and a 32-item FI [29] were previously operationalised and adapted to TILDA survey contents, and those adaptations were used in the present study.

Briefly, as per TILDA FP classification, individuals were considered to be frail when they met three or more of the following: measured slowness (based on timed up and go test), weakness (based on handgrip strength), and self-reported exhaustion, unintentional weight loss, and low physical activity. Non-frail referred to participants without any of those features [28].

The CFS was adapted to TILDA utilising the previously published classification tree, which takes into account recorded levels of help required with activities of daily living, overall number of chronic conditions, and self-reported general health, exhaustion (everything is an effort), and level of physical activity [30]. CFS scores were dichotomised into non-frail (1 to 3) and frail (5 to 9).

The 32 self-reported items composing the TILDA FI can be seen in Table A1. The FI expresses the proportion of deficits present in each participant out of the 32 deficits considered. Participants were dichotomised as non-frail (FI < 0.10) vs. frail (FI ≥ 0.25).

Regarding the intermediate category (pre-frail) for each frailty classification, rather than combining numbers with the non-frail or frail categories, they were excluded from the analyses as the primary focus was to compare established frailty vs. absence of frailty.

### 2.3. Active Stand 

The active standing test serves as a standardised method for evaluating a range of abnormal cardiovascular and neurovascular responses to the act of standing. Its purpose extends to assessing the underlying causes of orthostatic intolerance. In the TILDA wave 3 setup [31], six continuous non-invasive physiological signals were collected during the active standing test; three were collected in the cardiovascular domain, including systolic blood pressure (sBP), diastolic (dBP) blood pressure, and heart rate (HR), using a digital artery photoplethysmography device; and three were collected in the neurovascular domain, including frontal lobe cerebral oxygenation (Oxy), deoxygenation (Deoxy), and tissue saturation index (TSI), using near-infrared spectroscopy (NIRS). All measurements were carried out at an ambient temperature of 21 to 23 °C in a comfortably lit assessment room. Participants were instructed to lay in the supine position for ≈10 min before standing up and remain in the standing posture for 3 min afterwards, during which cardiovascular and neurovascular data were continuously recorded using the instrumentation detailed below. Participants were asked to stand up as swiftly as possible, and participants with mobility difficulties received assistance from a research nurse as needed.

### 2.4. Instrumentation

#### 2.4.1. Continuous Cardiovascular Signals

A Finometer device (Finometer MIDI, Finapres^®^ Medical Systems, Amsterdam, The Netherlands) was used to measure reconstructed arterial pressure noninvasively on a beat-to-beat basis. This is a photoplethysmography-based device that measures the pressure waveform of the finger arteries at 200 Hz using the volume-clamp method. The volume of the finger artery, which is measured by optical sensors embedded in the device, is maintained at a constant level throughout the assessment with the finger cuff actuated by a pneumatic control system [32]. Notably, the volume-clamp method has demonstrated robust agreements with both intra-arterial monitoring [33] and the auscultatory method [34]. The Finometer device also corrects for the hydrostatic height of the finger with respect to the heart level through a position sensor mounted to the finger.

#### 2.4.2. Continuous Neurovascular Signals

NIRS offers a non-invasive and non-ionising technology that has been employed for measuring variations in oxygenated and deoxygenated haemoglobin concentrations in diverse human tissues [35,36,37]. Studies have demonstrated the consistency of NIRS readings with other measurement modalities in various applications, such as cerebral blood flow [38] and skeletal muscle contractions [39]. NIRS’ versatility and high temporal resolution, facilitated by capabilities in time-resolved, frequency-domain, and continuous wave spectroscopic implementations, suggest its potential for a wide spectrum of applications in both research and clinical settings [40].

Based on optical sensing technology, NIRS measurements detect light absorbance across multiple wavelengths, with absorbance around 850 nm being attributed to oxyhaemoglobin (Oxy), and absorbance near 760 nm being ascribed to deoxyhaemoglobin (Deoxy) [41]. Combinations of Oxy and Deoxy are often reported, e.g., TSI expressed as 100 × Oxy/(Oxy + Deoxy) [42].

A wireless NIRS device, the PortaLite^®^ (Artinis Medical Systems, Elst, The Netherlands), was used to measure Oxy, Deoxy, and TSI signals, via absolute concentration method based on spatially resolved spectroscopy. With an optical sensor comprising an emitter and three receivers, the PortaLite^®^ has a capability of transmitting multi-channel, real-time data through Bluetooth^®^ at a maximum sampling frequency of 50 Hz. The user interface for the setup, recording, and export of NIRS data was facilitated using Oxysoft v3.0.53. The NIRS sensor was affixed approximately 2 cm above the left eye (approximately the FP1 (left frontal) position of the 10 to 20 electrode system (3 cm lateral and 3.5 cm superior to the nasion) [43], and the sampling frequency was set at 50 Hz for all participants. The influence of ambient light was minimised via a black headband covering the sensor [31].

### 2.5. Signal Acquisition, Synchronisation, and Preprocessing

This study focused on a one-minute segment of the active stand data, spanning from 20 s before the act of standing to 40 s after. The beat-to-beat cardiovascular signals from the Finapres^®^ MIDI were interpolated at 5 Hz. The neurovascular signals recorded using NIRS were downsampled to 5 Hz. All signals were synchronised via multiple manual markers throughout the recordings. The onset of the stand (i.e., the moment participants started standing up from the supine position) was determined via the height sensor data using an algorithm previously described in detail by O’Connor et al. [44]. Baseline cardiovascular and neurovascular values were established by averaging readings from 60 to 30 s prior to standing, in keeping with previous investigations [15,31,45].

### 2.6. Statistical Parametric Mapping

Statistical parametric mapping (SPM) is the application of Random Field Theory [46] to make a topological inference about whole-trajectory analysis. While its primarily application lies in neuroimaging [47], SPM can be applied to any signal that is a continuous function of space or time. For one-dimensional trajectories, like cardiovascular and neurovascular signals measured during the active standing test, SPM can be used to quantify the differences between multiple groups and identify the precise regions where significant differences are found in a temporal manner [21]. One-Dimensional Statistical Parametric Mapping (SPM1D) [48] is a Python/MATLAB package that has been employed to analyse various physiological traces [49,50,51]. Although several other software packages implementing the SPM methodology are readily available across various platforms (e.g., spmR, SPM12, and NIPY), SPM1D is currently the only package explicitly crafted for analysing one-dimensional data, such as the time series recorded during the active standing test [52].

### 2.7. Statistical Analyses

Descriptive statistics for the cohort and temporal analyses of the cardiovascular and neurovascular measures were carried out in R (version 4.0.5) using RStudio 2022.07.1+554 (Boston, MA, USA). The variables used for characterising the cohort included the following:

- Age and sex.
- Number of chronic conditions counted from a list comprising heart attack, heart failure, angina, cataracts, hypertension, high cholesterol, stroke, diabetes, lung disease, asthma, arthritis, osteoporosis, cancer, Parkinson’s disease, peptic ulcer, and hip fracture [53]. This information was further used to extract the list of cardiovascular diseases.
- Number of regular medications, excluding supplements.
- Number of physical limitations counted from a list that included walking 100 m (100 yards); running or jogging about 1.5 km (1 mile); sitting for about two hours; rising from a chair after sitting for long periods; climbing several flights of stairs without resting; climbing one flight of stairs without resting, stooping, kneeling, or crouching; reaching or extending arms above shoulder level; pulling or pushing large objects like a living room chair; lifting or carrying weights over 10 pounds/5 kilos (such as a heavy bag of groceries); and picking up a small coin from a table (Source: https://tilda.tcd.ie/data/documentation/; accessed on 10 November 2023).
- Baseline cardiovascular and neurovascular parameters.
- Self-reported dizziness during the entirety of the 3 min standing phase in the active standing test (yes or no).

For overall comparisons across groups, the independent-samples Kruskal–Wallis test and the Chi-squared test were used for non-normal continuous variables and categorical variables, respectively. Each of the three frail groups were also compared with the non-frail group using the unadjusted pairwise Wilcoxon rank-sum test. For these analyses, the statistical significance threshold was set at *p* < 0.05.

For SPM analyses, the open-source package SPM1d v0.4 (http://www.spm1d.org/, accessed on 1 November 2023), which is dependent primarily on SPM8 (https://www.fil.ion.ucl.ac.uk/spm/, accessed on 1 November 2023), was used in MATLAB environment (R2020b, The MathWorks, Inc., Natick, MA, USA). Independent *t* tests were conducted within SPM1d, which returns regions of significance in the form of *p* values. These values represent a continuous range over which the curve is identified as not consistent with random sampling. In order to reduce false positives and capture suprathreshold clusters [54] of likely clinical significance (i.e., continuous regions of at least 2 s of duration where at least one heartbeat would have been included), a statistical threshold of *p* < 0.001 was chosen.

## 3. Results

**Figure 1:**
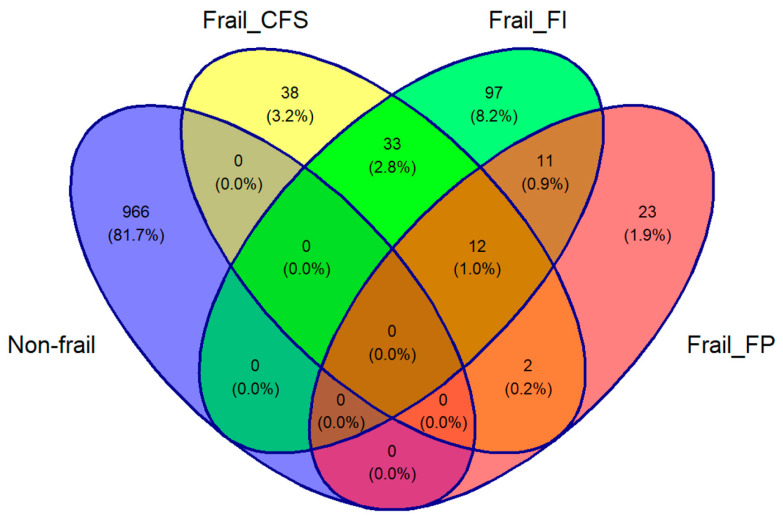
Venn diagram of the study population. The cohort was divided into three frail groups, including Clinical Frailty Scale (Frail_CFS), Frailty Index (Frail_FI), and Frailty Phenotype (Frail_FP). A fourth group was added including those who were classified as non-frail by all three criteria. Participants who were classified as frail by more than one classification, shown in the intersections in the Venn diagram, were excluded from the analysis.

There were 2133 participants aged ≥50 years in TILDA at wave 3 with complete Finometer and NIRS data. The overlaps between the frail groups and the non-frail group are depicted in Figure 1’s Venn diagram. Of this total of 1182 participants, 1124 (mean age of 63.5 years, 50.2% women) were included: 23 considered frail only by the FP, 97 by the FI, 38 by the CFS, and 966 by none.

The characterisation of all four groups included in the study is summarised in Table 1. As expected, all of the frail participants were more comorbid, more medicated, and more physically limited than the non-frail participants, but these differences were most accentuated for the FI classification. In the pairwise comparison, when compared to the non-frail group, only the group classified as frail by the FI exhibited a lower mean baseline Oxy (*p* = 0.015), higher baseline sBP (*p* = 0.037), and lower dBP (*p* = 0.008), and reported a significantly higher proportion of post-standing dizziness (*p* = 0.014). 

**Figure 2:**
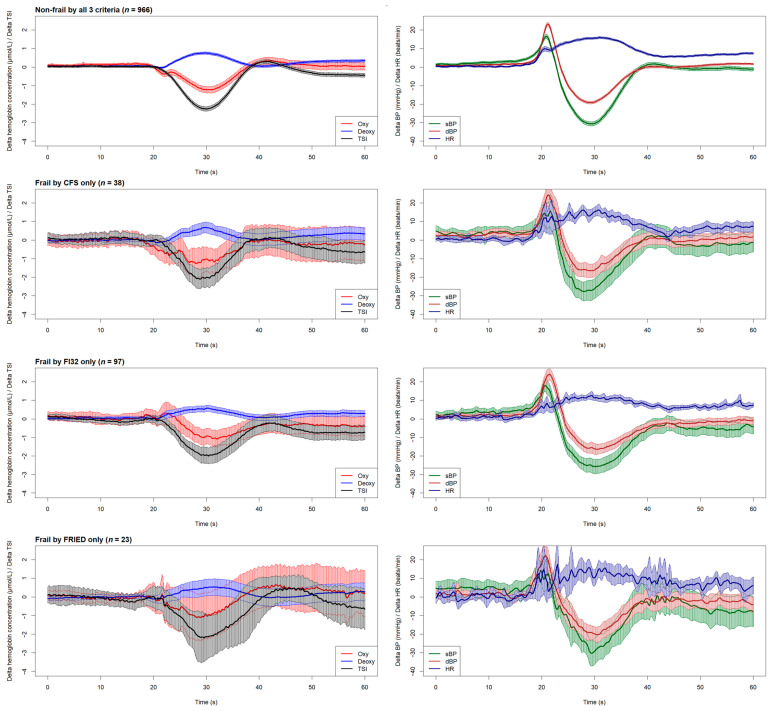
Time-series plots (mean with 95% CI) of the neurovascular signals (**left column**) including oxygenated haemoglobin (Oxy), deoxygenated haemoglobin (Deoxy), and tissue saturation index (TSI), and cardiovascular signals (**right column**) including systolic blood pressure (sBP), diastolic blood pressure (dBP), and heart rate (HR). All signals can be visually compared by different frailty groups, including non-frail participants (**top row**) and those classified as frail by different criteria (CFS, FI, and FP in rows 2, 3, and 4, respectively). Active standing starts at the 20 s mark in each plot. FI32: Frailty Index; CFS: Clinical Frailty Scale. FRIED indicates Frailty Phenotype (FP).

The graphical overview of cardiovascular and neurovascular signals shown in Figure 2 illustrates the physiological responses upon standing across all four groups included in this study.

**Figure 3:**
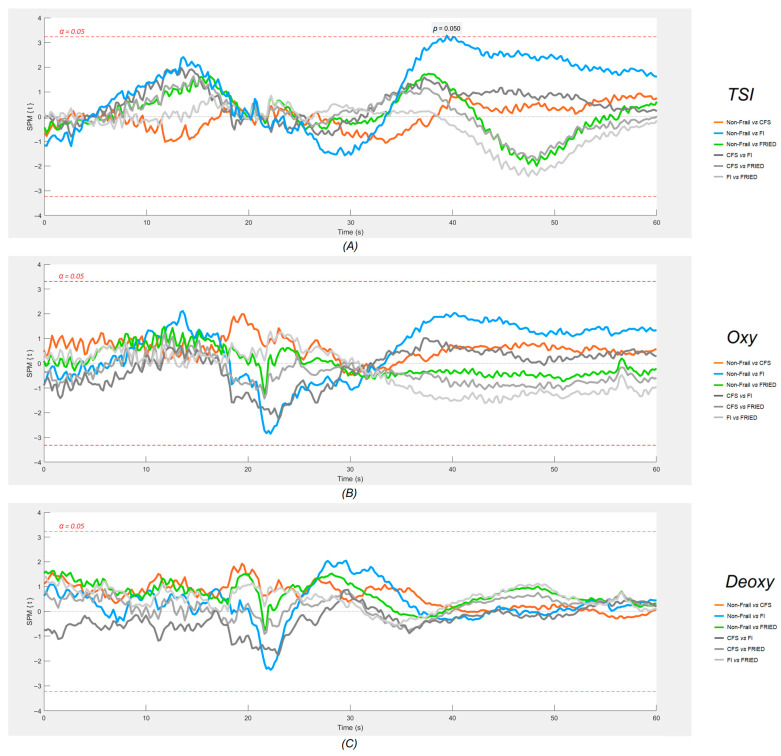
SPM plots of neurovascular signals ((**A**) tissue saturation index (TSI), (**B**) oxygenated haemoglobin (Oxy) and (**C**) deoxygenated haemoglobin (Deoxy)). The shaded regions that protrude over the dotted red lines are the locations where statistically significant (α = 0.05) differences were found between each frailty group and the non-frail group. Active standing starts at the 20 s mark on each plot. FI: 32-item Frailty Index; CFS: Clinical Frailty Scale. FRIED indicates Frailty Phenotype (FP).

**Figure 4:**
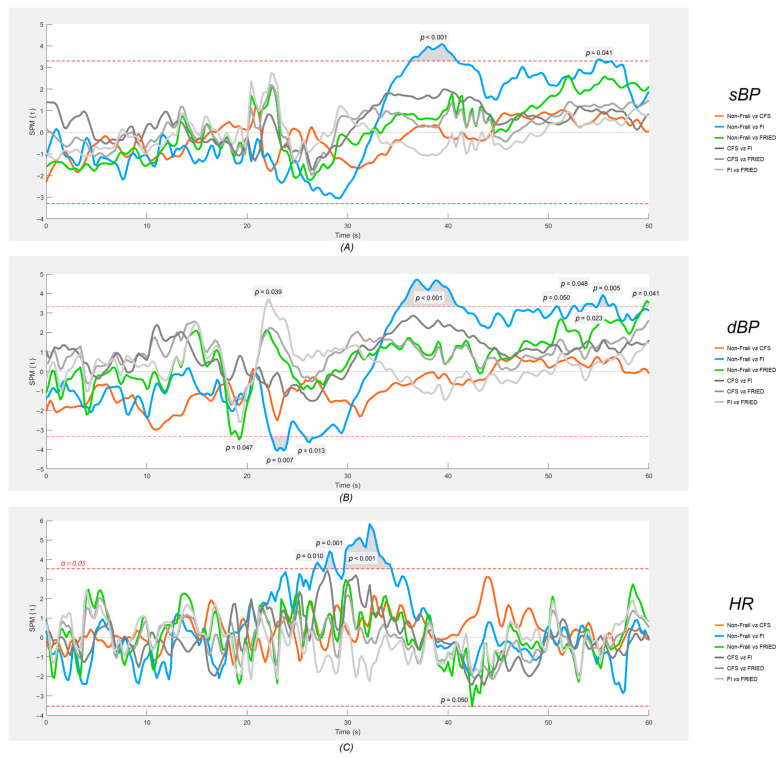
SPM plots of cardiovascular signals: (**A**) systolic blood pressure (sBP), (**B**) diastolic blood pressure (dBP), and (**C**) heart rate (HR). The shaded regions that protrude over the dotted red lines are the locations where statistically significant (α = 0.05) differences were found between each frail group and the non-frail group. Active standing starts at the 20 s mark on each plot. FI: 32-item Frailty Index; CFS: Clinical Frailty Scale. FRIED indicates Frailty Phenotype (FP).

Figure 3 (neurovascular) and Figure 4 (cardiovascular signals) show the results of the SPM analyses. As regards neurovascular signals (Figure 3), no significant differences were found at the *p* < 0.001 threshold.

As regards cardiovascular signals (Figure 4), the SPM analyses revealed that only the group that was considered frail by the FI had significantly different post-standing signals (*p* < 0.001) compared to the non-frail group, namely an attenuated gain in HR between approximately 10 and 15 s post-standing and larger deficits in both sBP and dBP between 15 and 20 s post-standing.

## 4. Discussion

The aim of this study was to compare frailty by three different identification criteria in their continuous cardiovascular and neurovascular responses to an active standing test, compared to the absence of frailty. We used data from wave 3 of TILDA and identified four mutually exclusive groups: groups considered frail only by the physical Frailty Phenotype (FP), the 32-item Frailty Index (FI), and the Clinical Frailty Scale (CFS) classification tree, and a fourth group where participants were not considered frail by any of these tools. As expected, all frail participants were more comorbid, more medicated, and more physically limited than the non-frail participants, but these differences were the most accentuated for the FI classification. In the pairwise comparison (Table 1), when compared to the non-frail group, only the group classified as frail by the FI exhibited a lower mean baseline Oxy, higher baseline sBP, and lower dBP, and reported a significantly higher proportion of post-standing dizziness. The SPM analyses revealed that only the group considered frail by the FI had significantly different signals compared to the non-frail group, namely an attenuated gain in HR between 10 and 15 s post-standing and larger deficits in sBP and dBP between 15 and 20 s post-standing.

As regards cardiovascular signatures, it is possible that frailty, encapsulating problems in multiple orthostatic compensatory mechanisms, may lead to a blunted primary cardiac pump response (indicated by a lesser increase in HR) in the initial post-standing period (10–15 s), leading to impaired blood pressure stabilisation during the early recovery period (15–20 s). Although this remains causally unproven, it has been seen across many studies [55,56,57,58,59,60,61,62,63,64]. Another interesting characteristic of our group that was considered frail by the FI is the fact that in the pairwise comparison analyses, it was the only frail group where the baseline sBP was significantly higher than that of the the non-frail group, which is reminiscent of the clinically challenging and risky syndrome of supine hypertension with concomitant orthostatic hypotension [65]. 

As regards neurovascular (NIRS) signals, the literature has much less information. A previous TILDA investigation conducted by Maguire et al. [21] pioneered the application of the one-dimensional SPM methodology to the cardiovascular and neurovascular comparisons of internal FI categories (i.e., non-frail, pre-frail, and frail) during the active standing test. Their study showed that a higher degree of FI frailty was associated with a lower orthostatic HR around 10 s post-stand and a lower TSI around 25 s post-stand. On the other hand, a clinical investigation by Perez-Denia et al. showed that multimorbidity in 303 falls in clinic attendees was associated with a poorer recovery of the TSI at 30 s after standing, as well as impaired dBP recovery at 30 s [66]. Even though in our study, the TSI difference between the FI and non-frail participants (which could have been transiently lower at 20 s post-stand, *p* = 0.05) did not reach our more stringent statistical significance threshold, the timing of this possible effect would be consistent with the findings of the previous two studies. This potential TSI signal may signify the activation of specific cerebral autoregulatory mechanisms by 20 s post-stand, which in the case of the frail by FI, might be less vigorous due to underlying cardiovascular/cerebrovascular disease.

Consequently, we hypothesise that the significant effect of the FI frailty classification and the lack of significant post-standing effects for the FP and CFS classifications may be due to the more direct capture of specific cardiovascular and neurovascular morbidities in the FI definition. This is evident in the cross-sectional characterisation of the frailty groups shown in Table 1, where the FI is the most dominant frailty criterion for discriminating the number of cardiovascular diseases. Crucially, the notable variations in the dynamics of neurocardiovascular signals among different frailty groups underscore the need for a more thorough investigation into the impact of each criterion used to classify frailty on physiological signals. Consequently, the insight gained from the present research emphasises the importance of recognising that diverse frailty classification criteria may exhibit distinct effects on physiological signals. Therefore, caution is advised in selecting the most suitable frailty assessment tools for specific research or clinical purposes. 

Technically, in this study, we demonstrated that plotting six continuous physiological signals in a synchronised fashion, as shown in Figure 2, provides not only an efficient way to visually inspect the physiological responses of different groups during active standing, but also makes it possible to postulate possible connections between cardiovascular and neurovascular responses, allowing for the generation of hypotheses to be tested in further studies. The results of the one-dimensional SPM analyses suggested that this is quite a sensitive tool for the detection of signal differences, as judged by very minor and transient pre-standing differences that are unlikely to be of clinical significance (e.g., Non-Frail vs. FRIED and FI vs. FRIED differences in dBP, as seen in Figure 4). In this regard, the post-standing TSI difference FI vs. non-frail seemed modest in comparison with the more obvious and temporally sustained sBP and dBP signal differences.

In various fields, it is common practice to filter raw data recorded from physiological assessments before conducting formal analyses. While adequate data filtering can boost processibility in data analyses [67], enhance the visual clarity of the graphical results [68], and lower the cost of storing and maintaining the database [69], it could lead to over-processing, therein, the application of excessive filtering results in unwanted data loss that could hold pathophysiological and/or clinical significance [70,71]. In this study, all physiological signals were analysed without being filtered. Despite the noticeably jagged trajectories of each signal on the plots, as opposed to a much smoother finish in a previous SPM study conducted by Maguire et al. [21], in which moving average and median filters were employed, the analysis of unfiltered data unmistakably depicted the trends in physiological responses within the designated timeframe (as shown in Figure 2, Figure 3 and Figure 4) for both cardiovascular and neurovascular signals. Above all, the proposed methods effectively captured the intricacies of every signal at the utmost resolution, enabling comprehensive examinations of the interconnected response within neurovascular and cardiovascular signals subjected to an orthostatic challenge with remarkable precision. 

To our knowledge, our study is the first to utilise the SPM methodology to compare more than one frailty measure across active standing dynamics in mutually exclusive groups. Another strength of our design is the large population-based sample from which the physiological data were collected, although the need for mutually exclusive groups and the exclusion of pre-frail groups to maximise non-frail vs. frail differences reduced the size of the study population. This precluded a subanalysis by sex, which could be of potential interest [66]. Finally, another limitation of the study is that our FP definition in TILDA is an adaptation of the original criteria, and the CFS is based on a retrospective classification tree rather than contemporaneous face-to-face scoring in TILDA participants. Self-report limitations may also apply to the frailty tools, and it is to be noted that the 32-item TILDA FI was entirely self-reported. We also acknowledge that while TILDA offers insights into the Irish community-dwelling context, it is important to replicate the research in various settings and countries to enhance the external validity of our findings.

## 5. Conclusions

In our analysis, different frailty identification tools captured different continuous cardiovascular and neurovascular responses to an orthostatic stress test. The FI had better discrimination than FP and CFS possibly because of the better capture of cardiovascular morbidities, and it may therefore have better clinical applicability. As a pioneering study in the applicability of multiple established frailty tools to non-invasively collected neurocardiovascular signals, we captured significant differences in the dynamics of the signals among the frail population identified by different frailty criteria, shedding light on the awareness that different frailty classification criteria can render differently on physiological signals and the necessity of considering the applicability of frailty identification tools when physiological signals are the subject of investigation. 

## Figures and Tables

**Table 1:** Characterisation of the groups investigated in this study. SD: standard deviation; sBP: systolic blood pressure; dBP: diastolic blood pressure; HR: heart rate; bpm: beats per minute; TSI: tissue saturation index; Oxy: oxygenated haemoglobin; Deoxy: deoxygenated haemoglobin; CFS: clinical frailty scale; FI: frailty index; FP: frailty phenotype.

Characteristic	Non-Frail	Frail-CFS	Frail-FI	Frail-FP	*p* _Overall_	*p* _CFS-NonFrail_	*p* _FI-NonFrail_	*p* _FP-NonFrail_
Mean age ± SD (years)	62.7 ± 6.4	64.1 ± 6.4	69.4 ± 7.6	70.6 ± 9.8	≤0.001	≤0.001	≤0.001	≤0.001
Male sex (%)	0.5	0.6	0.4	0.6	0.020	0.600	0.004	0.750
Mean number of chronic diseases	0.7	1.9	3.1	1.8	≤0.001	≤0.001	≤0.001	≤0.001
Mean number of cardiovascular diseases	0.6	0.9	2.3	1.3	≤0.001	0.038	≤0.001	≤0.001
Mean number of regular medications	1.1	2.5	5.7	3.3	≤0.001	≤0.001	≤0.001	≤0.001
Mean number of physical limitations	0.8	3.6	4.9	2.1	≤0.001	≤0.001	≤0.001	≤0.001
Mean baseline sBP (mmHg)	139.7	140.1	144.8	142.1	0.178	0.667	0.037	0.454
Mean baseline dBP (mmHg)	76.8	74.3	74.1	74.6	0.031	0.148	0.008	0.505
Mean baseline HR (bpm)	64.8	64.7	67.1	69.8	0.043	0.939	0.103	0.016
Mean baseline TSI (%)	71.9	71.5	71.6	71.2	0.782	0.610	0.390	0.680
Mean baseline Oxy (µmol/L)	29.3	26.7	26.9	27.7	0.038	0.125	0.015	0.352
Mean baseline Deoxy (µmol/L)	10.9	10.1	10.2	10.3	0.186	0.171	0.087	0.529
Dizziness during the active stand (%)	25.5	31.6	37.1	21.7	0.078	0.402	0.014	0.681

## Data Availability

The datasets generated and/or analysed during the current study are not publicly available due to data protection regulations but are accessible at TILDA upon reasonable request. The procedures to gain access to TILDA data are specified at https://tilda.tcd.ie/data/accessing-data/ (accessed on 1 November 2023).

## Appendix A

**Table A1:** Frailty Index with 32 items: items and scoring of individual items.

Self-Reported Deficit	Scoring
Difficulty walking 100 m	0 = No; 1 = Yes
Difficulty rising from a chair	0 = No; 1 = Yes
Difficulty climbing one flight of stairs	0 = No; 1 = Yes
Difficulty stooping, kneeling or crouching	0 = No; 1 = Yes
Difficulty reaching above shoulder height	0 = No; 1 = Yes
Difficulty pushing/pulling large objects	0 = No; 1 = Yes
Difficulty lifting/carrying weights ≥ 10 pounds (4.5 kg)	0 = No; 1 = Yes
Difficulty picking up a coin from a table	0 = No; 1 = Yes
Feeling lonely	0 = None of the time, rarely; 0.5 = Some of the time; 1 = All the time
Self-rated physical health	0 = Excellent, Very good, Good; 0.5 = Fair; 1 = Poor
Self-rated vision	0 = Excellent, Very good, Good; 0.5 = Fair; 1 = Poor
Self-rated hearing	0 = Excellent, Very good, Good; 0.5 = Fair; 1 = Poor
Self-rated day-to-day memory	0 = Excellent, Very good, Good; 0.5 = Fair; 1 = Poor
Difficulty following a conversation with one person	0 = None; 0.5 = Some; 1 = Much/Impossible
Daytime sleepiness	0 = Never, slight chance; 0.5 = Moderate chance; 1 = High chance
Polypharmacy	0 = No; 1 = Yes
Knee pain	0 = No; 1 = Yes
Hypertension	0 = No; 1 = Yes
Angina	0 = No; 1 = Yes
Heart attack	0 = No; 1 = Yes
Diabetes	0 = No; 1 = Yes
Stroke or Transient ischaemic attack	0 = No; 1 = Yes
High cholesterol	0 = No; 1 = Yes
Irregular heart rhythm	0 = No; 1 = Yes
Other cardiovascular disease	0 = No; 1 = Yes
Cataracts	0 = No; 1 = Yes
Glaucoma or Age-related macular degeneration	0 = No; 1 = Yes
Arthritis	0 = No; 1 = Yes
Osteoporosis	0 = No; 1 = Yes
Cancer	0 = No; 1 = Yes
Varicose ulcer	0 = No; 1 = Yes
Urinary incontinence	0 = Never, slight chance; 0.5 = Moderate chance; 1 = High chance
